# Virus-induced asthma attack: The importance of allergic inflammation in response to viral antigen in an animal model of asthma

**DOI:** 10.1371/journal.pone.0181425

**Published:** 2017-07-24

**Authors:** Christopher Skappak, Ramses Ilarraza, Ying-qi Wu, Matthew G. Drake, Darryl J. Adamko

**Affiliations:** 1 Division of Emergency Medicine, McMaster University, Hamilton, Ontario, Canada; 2 Departments of Pediatrics, University of Alberta, Edmonton, Alberta, Canada; 3 Division of Pulmonary and Critical Care Medicine, Oregon Health & Science University, Portland, Oregon, United States of America; 4 Department of Pediatrics, University of Saskatchewan, Saskatoon, Saskatchewan, Canada; University of Tennessee Health Science Center, UNITED STATES

## Abstract

Asthma exacerbation can be a life-threatening condition, and is most often triggered by common respiratory viruses. Poor asthma control and worsening of respiratory function is associated with increased airway inflammation, including eosinophilia. Prevention of asthma exacerbation relies on treatment with corticosteroids, which preferentially inhibit allergic inflammation like eosinophils. Human studies demonstrate that inactivated virus can trigger eosinophil activation *in vitro* through antigen presentation and memory CD4+ lymphocytes. We hypothesized that animals with immunologic memory to a respiratory virus would also develop airway hyperresponsiveness in response to a UV-inactivated form of the virus if they have pre-existing allergic airway inflammation. Guinea pigs were ovalbumin-sensitized, infected with live parainfluenza virus (PIV), aerosol-challenged with ovalbumin, and then re-inoculated 60 days later with live or UV-inactivated PIV. Some animals were either treated with dexamethasone prior to the second viral exposure. Lymphocytes were isolated from parabronchial lymph nodes to confirm immunologic memory to the virus. Airway reactivity was measured and inflammation was assessed using bronchoalveolar lavage and lung histology. The induction of viral immunologic memory was confirmed in infected animals. Allergen sensitized and challenged animals developed airway hyperreactivity with eosinophilic airway inflammation when re-exposed to UV-inactivated PIV, while non-sensitized animals did not. Airway hyperreactivity in the sensitized animals was inhibited by pre-treatment with dexamethasone. We suggest that the response of allergic inflammation to virus antigen is a significant factor causing asthma exacerbation. We propose that this is one mechanism explaining how corticosteroids prevent virus-induced asthma attack.

## Introduction

The most common trigger for asthma exacerbation in adults and children, are infection with rhinovirus, though other common respiratory viruses, like parainfluenza virus, cause asthma exacerbation [1–3]. The reason for this severe response to the common cold is unclear.

Exposure and re-exposure to respiratory viruses seasonally is a common occurrence, one that allows for the natural development of an adaptive immune response to the virus [4, 5]. There are various phenotypes of asthma, the most prevalent being allergic or atopic asthma [6]. One of the proposed causes for viral induced exacerbations in patients with allergic asthma is the degree of chronic allergic inflammation found in their airways prior to virus infection [7]. Being atopic and having associated airway eosinophilia represents the strongest predictor of hospitalization in relation to virus infection [8]. Eosinophils have been well described as vectors for airway dysfunction when activated [9]. The cornerstone of asthma therapy, corticosteroids, are especially effective against allergic Th_2_ type inflammation and the associated eosinophils [10].

In the context of respiratory virus infection, *in-vivo* studies have demonstrated that eosinophils have a direct role in causing airway hyperresponsiveness, a mechanism that appears to require lymphocyte responses to the virus [11] [12, 13]. *In-vitro*, eosinophils can be activated by virus, but only after co-culture with lymphocytes[12]. Even UV-inactivated virus (i.e., non-replicating viral particles) can induce eosinophil activation through CD4+ memory lymphocytes [12].

Throughout their lives, individuals encounter similar strains of respiratory virus multiple times facilitating the development of virus specific immunologic memory. Given the *in vitro* data, we hypothesized that the eosinophilic inflammation present in the airways prior to viral infection could be an important factor in the development of airway hyperreactivity (AHR) and not just the severity of viral infection. If immunologic memory to a virus were in place, this allergic inflammation could be activated by exposure and presentation of non-infectious viral particles circulating as inert antigens. To test this, we developed a guinea pig model that has both viral immunologic memory and allergic airway inflammation. For the first time *in-vivo*, we demonstrate that allergic inflammation can be triggered to induce airway dysfunction through exposure to a non-replicating virus to which immunologic memory already exists. AHR did not develop in non-allergic animals even though they had also developed immunologic memory and were exposed to the non-replicating virus. We suggest that this model better reflects real life responses in atopic and non-atopic humans with repeated local environmental exposure to common respiratory viruses. It also highlights the importance of allergic immune response to viruses rather than just the degree of virus infection as a cause of asthma attack.

## Results

### Experimental protocol

**Fig 1:**
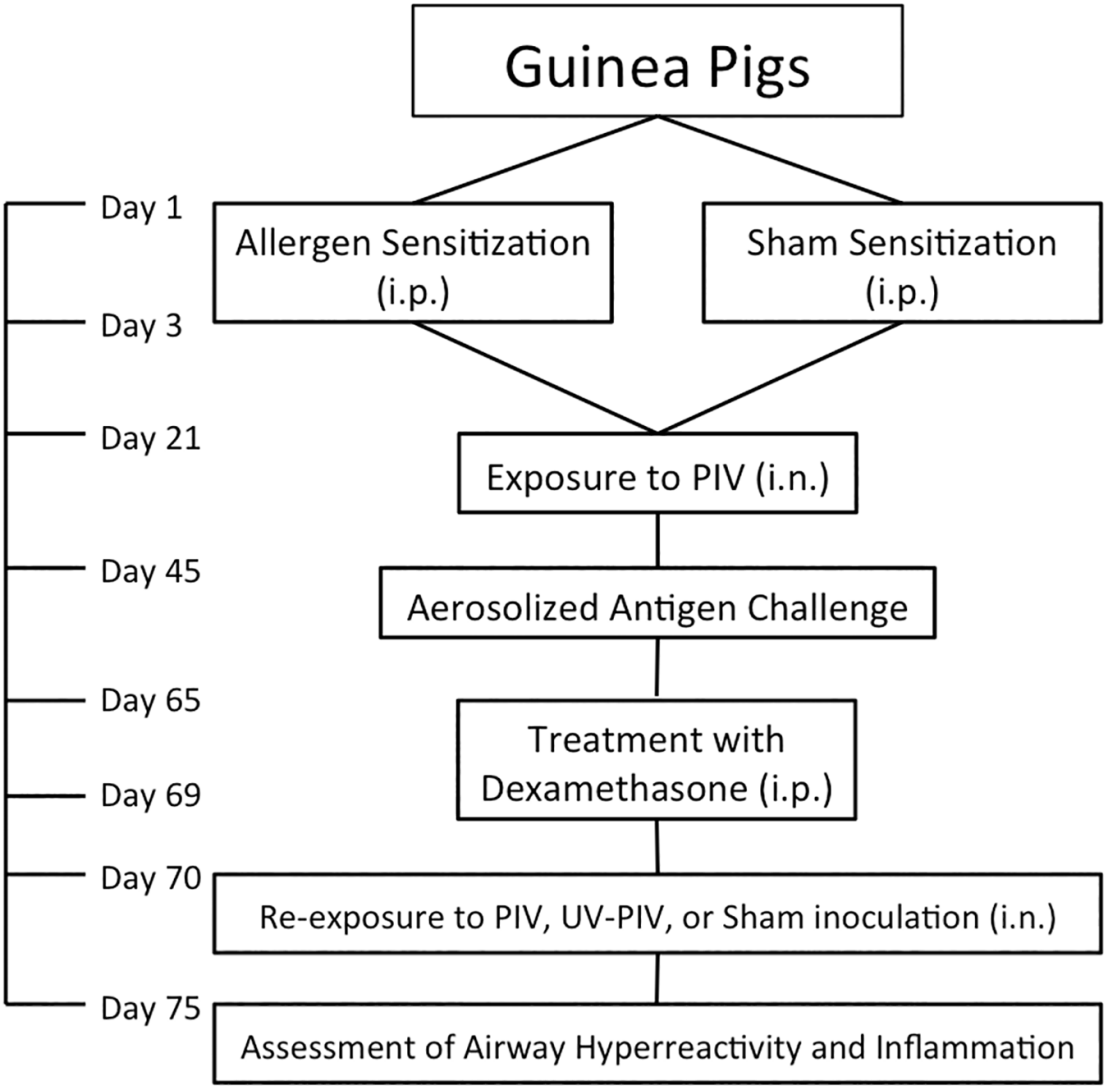
Experimental timeline flowchart. Guinea pigs were sensitized to ovalbumin or given sham sensitizations via i.p. injections on Days 1 and 3. Sensitized and non-sensitized GPs were inoculated intranasally with parainfluenza virus (PIV) on Day 21. After recovery from virus or sham infection, all GPs were exposed to an aerosolized solution of ovalbumin on Day 45, with the exception of age-matched controls. Some GPs received dexamethasone on Days 61 to 65. Animals were inoculated intranasally with live-PIV, UV-PIV, or sham (NaCl 0.9%) on Day 70. On Day 75 all animals underwent airway hyperreactivity and inflammation assessment.

Guinea pigs involved in this study followed an experimental time line beginning 2 weeks after animals arrived at the facility (Fig 1). The timeline of experiments is based on previous publications using these animals and these interventions. On Days 1 and 3 some animals were sensitized to ovalbumin via i.p injections. Non-sensitized animals were given sham i.p. injections on the same timeline. On Day 21 some animals in both groups were then inoculated intranasally with PIV. Animals were allowed to recover until Day 45 when they were exposed to an inhaled ovalbumin allergen challenge. From Day 65 to 69 some sensitized and non-sensitized were treated with daily i.p. injections of dexamethasone or sham i.p. injections with NaCl 0.9%. All animal groups underwent exposure to intranasal PIV, UV-PIV, or sham inoculation with NaCl 0.9%. On Day 75 all animals underwent assessment of airway hyperreactivity and were euthanized following the experiments. Age matched controls (sensitized or not) were housed in the same facility for the duration of the study. They received NaCl 0.9% sham treatments on the same timeline as the other animals.

### Confirmation of immune memory to parainfluenza virus (PIV)

**Fig 2:**
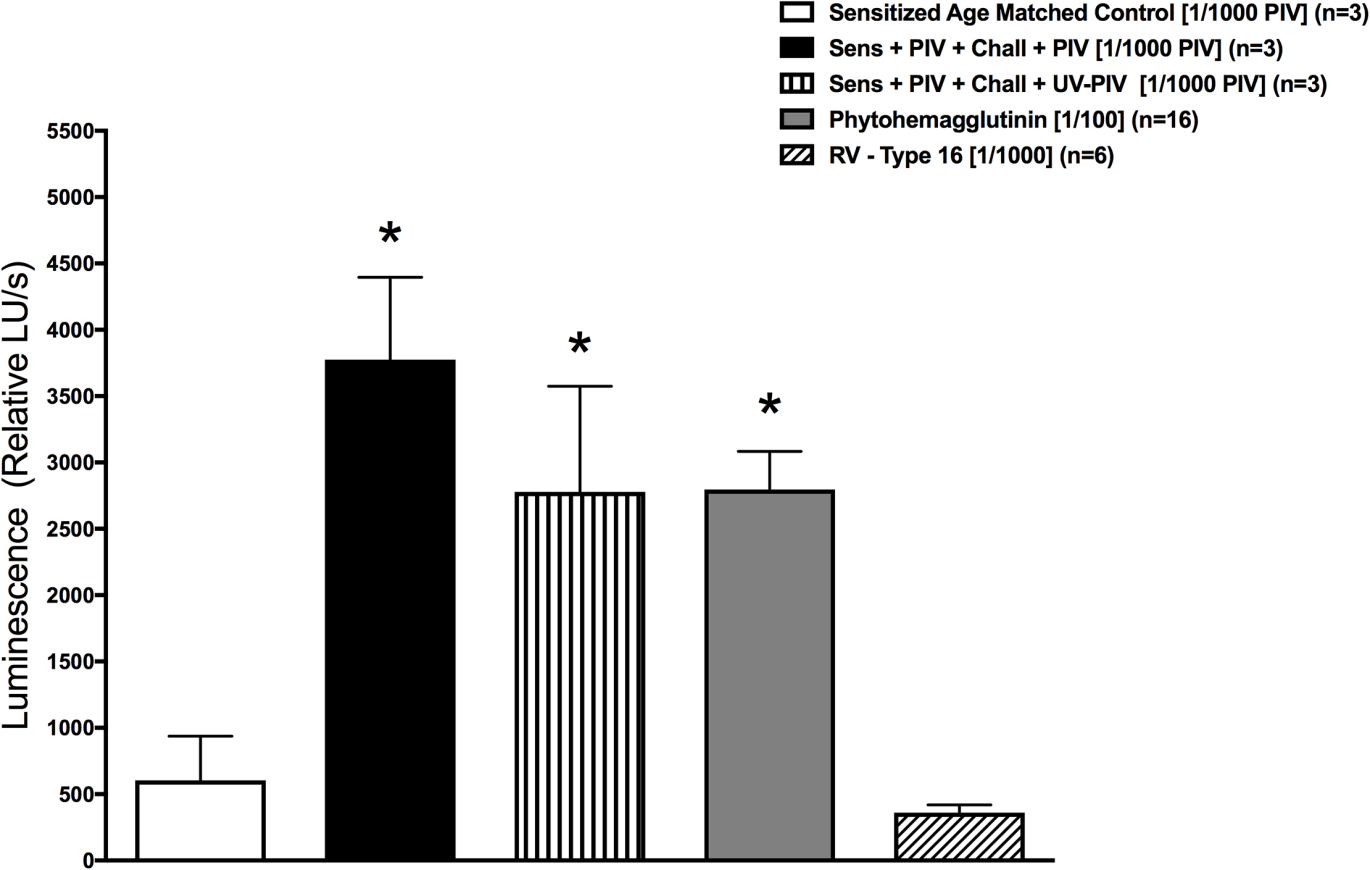
Enriched lymphocyte cell cultures demonstrated virus specific proliferation when immune memory was present. Animals re-infected with live PIV (black bar) and inoculated with UV-inactivated PIV (UV-PIV, vertical striped bar) exhibited BrdU incorporation when exposed to PIV *in-vitro* (n = 3, p<0.001 and n = 3, p<0.01 respectively). The level of proliferation was similar to the positive control stimulated with phytohemagglutinin (gray bar) (n = 16, p<0.01), and all were significantly higher than the uninfected age matched controls (white bar) (n = 3). Exposure to a different virus, Rhinovirus (RV) type-16 (n = 6), did not induce BrdU incorporation. Error bars represent SD.

Lymphocytes from sensitized PIV infected animals cultured *in vitro* with a 1x10^4^ TCID_50_/mL dose of PIV stock solution showed significant proliferation, compared to cells from non-infected age-matched control animals lacking immune memory (n = 3, p<0.001 and n = 3, p<0.01 respectively) (Fig 2). In addition, the data suggest that this response was due to specific memory to PIV, as lymphocytes from these animal groups did not show proliferation in response to an unrelated respiratory virus, Rhinovirus-16.]

### Re-infection with live parainfluenza virus induces airway hyperreactivity and airway inflammation in both non-sensitized and sensitized animals

**Fig 3:**
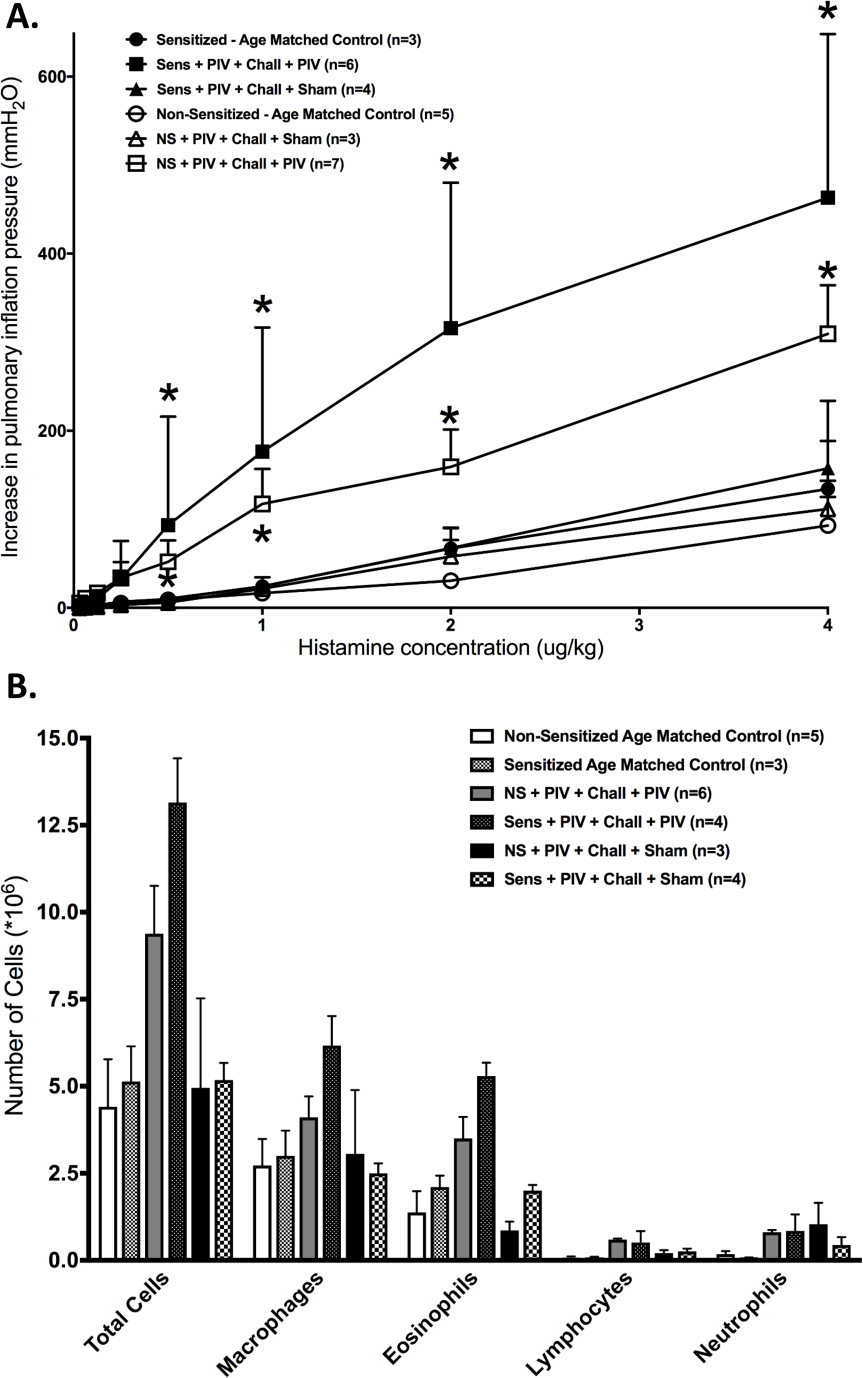
Repeat exposure to live virus induces airway hyperreactivity and airway inflammation in both non-sensitized and sensitized animals. **(A)** Both non-sensitized and sensitized animals had significantly higher bronchoconstriction in response to histamine (i.v.) after re-infection with live PIV (closed square, n = 5 each), when compared to respective uninfected sham inoculated controls (open triangle, n = 3 each) or age-matched controls (n = 5 each; p<0.0001 each). **(B)** Non-sensitized animals re-exposed to live PIV (n = 6) have significantly higher total cell counts in the BAL compared to age-matched (n = 5) and sham controls (n = 3; p<0.001 for both). Sensitized animals re-infected with live PIV (n = 4) also developed higher total cell counts in the BAL compared to age-matched (n = 3) and sham controls (n = 4; p<0.001 for each). In the sensitized animals, macrophages and eosinophils were significantly more abundant. Error bars represent SEM.

As it would be expected, both non-sensitized and sensitized GPs that were re-exposed to live PIV on day 70 showed a significant increase in airway reactivity to histamine compared to respective age matched and sham-infected controls (Fig 3A). The degree of increased bronchoconstriction was higher in the sensitized GP compared to non-sensitized GP. Both non-sensitized and sensitized GP re-exposed to live PIV showed statistically significantly increases in total cell numbers in the BAL compared to their respective uninfected controls (Fig 3B). In regard to individual cell types, macrophages and eosinophils were significantly different when comparing in the sensitized animals compared to sham controls (Fig 3B).

### Inoculation with UV-inactivated parainfluenza virus induces airway hyperreactivity, but only in sensitized animals

**Fig 4:**
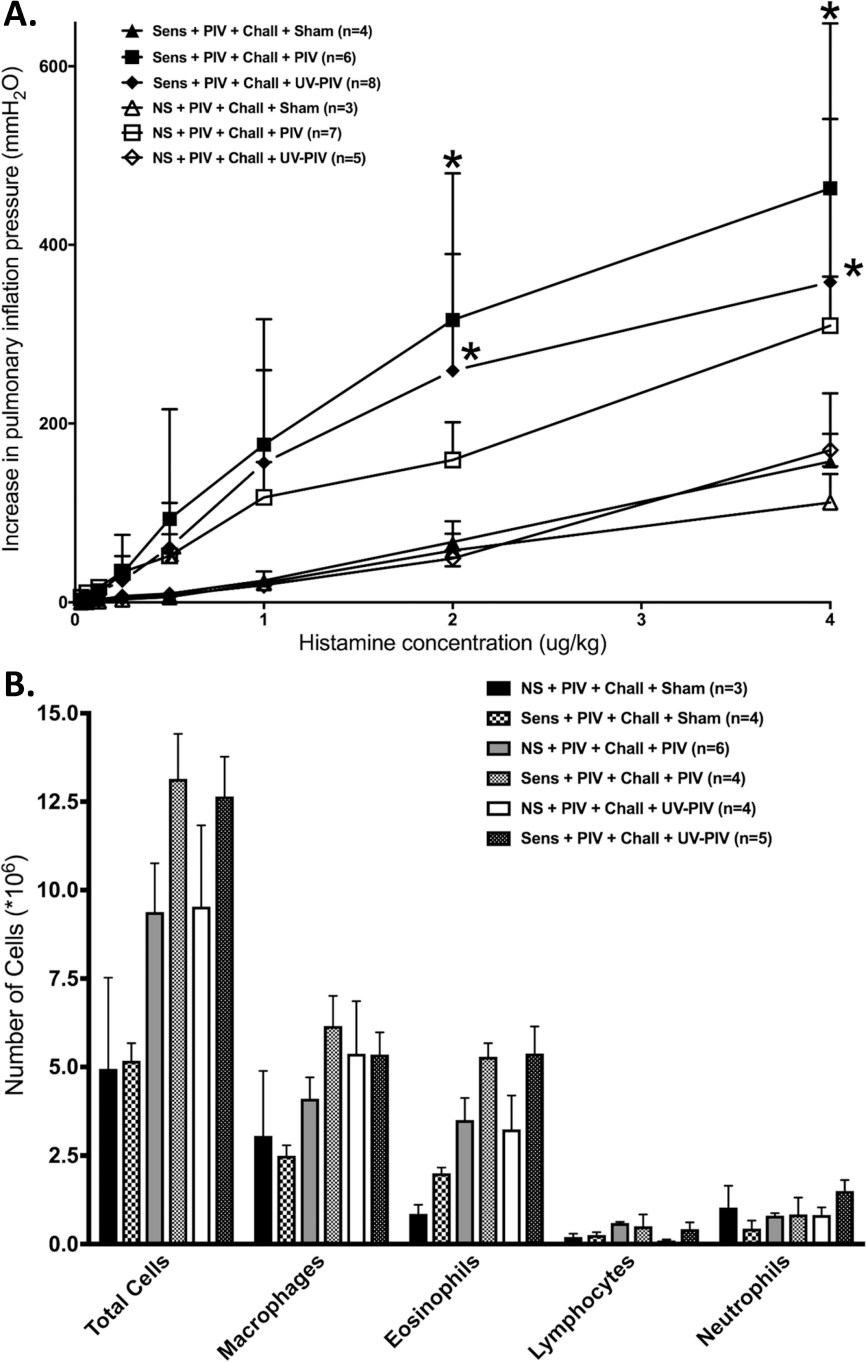
Inoculation with UV-inactivated virus induced airway hyperreactivity in only sensitized animals. **(A)** Non-sensitized animals inoculated with UV-PIV (n = 5; p<0.001), had significantly lower bronchoconstriction in response to histamine (i.v.) compared to animals infected with live virus (n = 5). In contrast, sensitized animals inoculated with UV-inactivated virus (closed diamond, n = 8) showed high levels of bronchoconstriction in response to histamine (i.v.) similar to animals re-infected with live-PIV (n = 5). Sham inoculated controls were also included (open triangle, n = 5 each). **(B)** Non-sensitized animals inoculated with UV-PIV (n = 5) showed a significant increase in total cell numbers in the BAL compared to sham controls (n = 3; p = 0.05). Differences in individual cell types did not reach statistical significance. Sensitized animals inoculated with UV-PIV (n = 5; P<0.0001) showed significantly higher total cell numbers, with higher macrophage and eosinophil cell counts, compared to sham controls (n = 4; P<0.01 and P<0.01 respectively). Error bars represent SEM.

Some non-sensitized animals with immune memory were inoculated at day 70 with UV-inactivated PIV, instead of infectious PIV. These animals did not develop increased reactivity to histamine compared to animals that were re-exposed to live PIV or the uninfected controls (Fig 4A). In contrast, sensitized GPs with immune memory and re-exposure to UV-PIV exhibited a significant increase in airway reactivity compared to sham controls. Both non-sensitized and sensitized UV-PIV inoculated animals had significantly increased total cell numbers in the BAL compared to their respective sham controls (Fig 4B). In regard to individual cell types, only the presence of macrophages and eosinophils in the sensitized animals was significantly higher compared to respective sham controls.

### Treatment with dexamethasone prior to secondary live PIV infection prevents airway hyperreactivity only in sensitized animals

**Fig 5:**
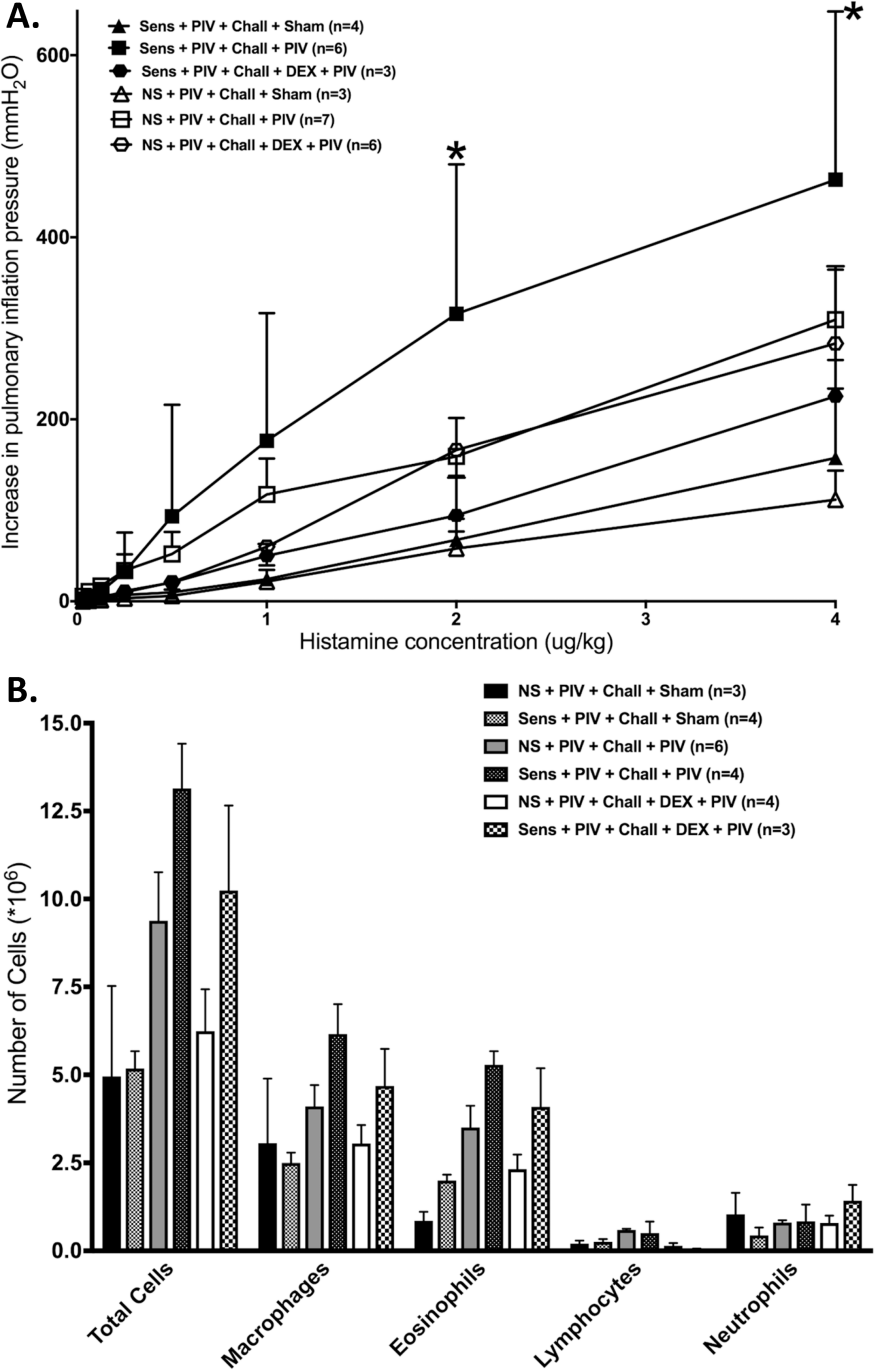
Treatment with dexamethasone prior to secondary live virus infection prevents airway hyperreactivity only in sensitized animals. Some animals were treated with dexamethasone (i.p.) before the second live PIV infection. **(A)** Dexamethasone treatment of non-sensitized animals before secondary infection with PIV (n = 6) had no effect on bronchoconstriction in response to histamine (i.v.), compared to untreated animals (n = 5). In contrast, in OVA-sensitized animals, dexamethasone treatment (n = 3) significantly reduced bronchoconstriction responses (n = 4; p = 0.001). Sham inoculated controls were also included (n = 5 each). **(B)** Dexamethasone treatment before live PIV re-infection (white bars, n = 4, p<0.01) induced a significant decrease in total cell numbers compared to untreated and re-infected non-sensitized and sensitized animals (n = 6: p<0.01), but this did not reach statistical significance for individual cell counts. Error bars represent SEM.

Dexamethasone treatment prior to infection with live PIV had no significant effect on airway reactivity of non-sensitized animals (Fig 5A). In contrast, in animals that were sensitized with OVA and treated with dexamethasone before live PIV infection, there was a significant decrease in airway reactivity. Both non-sensitized and sensitized animals treated with dexamethasone before live PIV infection showed significantly decreased total cell numbers in the BAL, when compared to untreated PIV infected animals, but no statistical significance was seen in the individual cell counts (Fig 5B).

### Treatment with dexamethasone before inoculation with UV-inactivated virus prevents airway hyperreactivity and airway inflammation in sensitized animals

**Fig 6:**
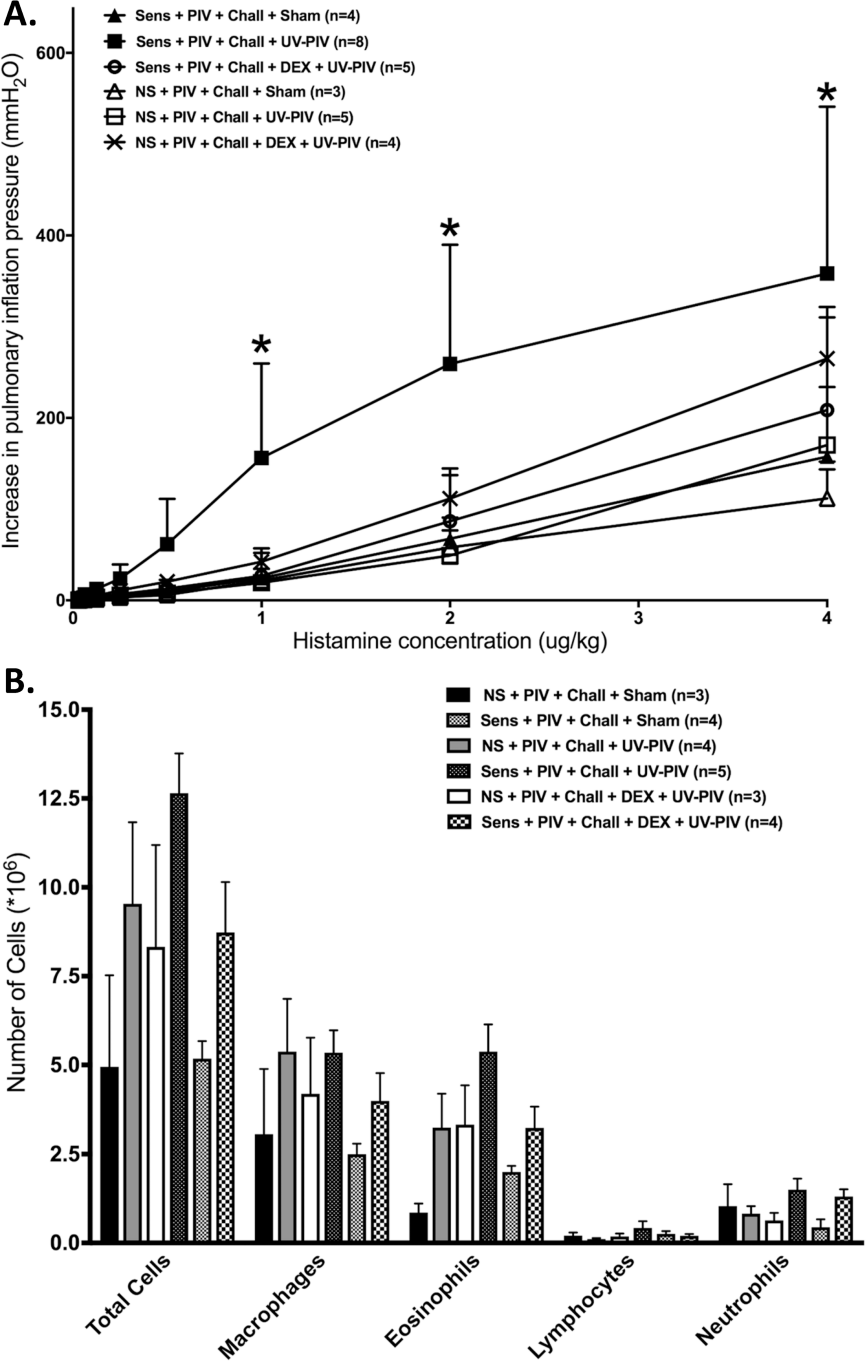
Treatment with dexamethasone before inoculation with UV-inactivated virus prevents airway hyperreactivity in sensitized animals. **(A)** Non-sensitized animals treated with dexamethasone before UV-PIV inoculation (n = 5) showed higher bronchoconstriction response compared to sham controls (n = 3) and untreated UV-PIV animals (n = 5; p<0001). In contrast, sensitized animals treated with dexamethasone prior to UV-PIV inoculation (n = 5) showed significantly lower bronchoconstriction responses compared to the untreated animals inoculated with UV-PIV (n = 8; p<0.0001). Also shown are sham inoculated controls (n = 5 each). **(B)** Non-sensitized animals treated with dexamethasone before UV-PIV (white bar, n = 3) did not show significant changes in BAL compared to untreated UV-PIV-exposed animals (n = 4); in contrast, dexamethasone-treated animals exposed to UV-PIV (n = 4) showed significantly decreased total cell numbers compared to untreated UV-PIV (n = 5; p = 0.05). Total cell counts also remained significantly higher compared to sham controls (n = 4; p<0.0001). Error bars represent SEM.

Non-sensitized animals treated with dexamethasone before inoculation with UV-PIV showed a small but significant increase in airway reactivity compared to untreated animals exposed to UV-PIV alone (Fig 6A). In contrast, sensitized GPs treated with dexamethasone before inoculation with UV-PIV demonstrated a significant decrease in airway reactivity compared to untreated PIV-infected animals, similar to uninfected controls. Total cell numbers and individual cell counts did not show significant differences in the BAL of non-sensitized animals treated with dexamethasone before UV-PIV inoculation (Fig 6B). In contrast, treatment with dexamethasone before UV-PIV inoculation resulted in a decrease in the total cell number in the BAL of sensitized animals, albeit differences did not reach statistical significance amongst individual cell types.

## Discussion

Corticosteroid therapy, which decreases allergic airway inflammation, is the backbone of asthma management [14]. Corticosteroid medications are so far the most effective method to prevent asthma exacerbations [15, 16]. The majority of asthma attacks are associated with respiratory viral infection, via mechanisms still poorly understood. It is well accepted that being an atopic person is one of the strongest factors predicting asthma exacerbation upon respiratory virus infection in humans [17]. We hypothesized that the presence of allergic inflammation before virus infection is an important cause of severe airway dysfunction and not just the degree of infection. For the first time, we provide evidence that viral antigen without infection can trigger an allergic inflammatory response and induce airway hyperreactivity, *in-vivo* when immunological memory is present.

Another common hypothesis for virus-induced asthma attack centers on viral load, severity of infection and the inability of atopic patients to mount a proper immunological response to the infection [18–20]. Other human studies have called into question the former hypothesis regarding viral load. Using a common human respiratory pathogen, Rhinovirus, Bardin *et al*., *Kennedy* et al., and Denlinger *et al*. found that atopic and non-atopic human subjects had similar levels of viral burden and viral shedding [21, 22], [19]. Further work by Bardin *et al*. confirmed that atopic patients have increased levels of allergic inflammation in their upper respiratory tract [21]. Our findings further support the hypothesis that the severity of the viral infection may not be the sole cause of virus-induced asthma exacerbation in atopic patients.

Studies in both human and animal models of asthma have identified the role of eosinophils, and their relevance to virus-induced asthma exacerbations. There is substantial evidence that eosinophil degranulation products released into the airways following viral infections directly correlate with airway damage and worsening asthma [23, 24]. Sputum eosinophilia has been demonstrated to correlate with the severity of airway dysfunction in asthmatics [25, 26]. Our previous work in allergen sensitized animal models with primary PIV infection, demonstrated that viral induced airway hyperreactivity can be mediated by eosinophils [11]. As would be expected in a primary infection model, we demonstrated that CD8+ T-cells were key in the activation of this eosinophil mediated pathway after viral infection [27]. We went on to show that viral immunologic memory in humans, mediated via memory CD4+ T cells, could induce eosinophil activation *in-vitro* when these cells were presented with a virus [28]. These *in-vitro* human and *in-vivo* findings were the basis to study whether immunologic memory to common respiratory pathogens could cause airway dysfunction in allergic airways.

Most respiratory virus studies focus on the primary infection by the virus. However, humans are continuously exposed and re-exposed to airway viruses, a feature that is absent in primary infection models. Few models have included the effect of secondary exposure to virus. One based on neonatal mice, was designed to assess the effects of early life viral infections and long-term immunological changes [29, 30]. Unfortunately, that study did not examine inflammation prior to infection, nor had they attempted to discern the mechanism behind the virus induced AHR. However, their results corroborate our previous findings that a Th_2_ phenotype alters severity of AHR caused by respiratory viral infection. Furuya et al. described the effects of repeated infections of different strains of influenza in allergic mice in order to understand how cross immunity is different in asthma [31]. They reported that cross protection was impaired in asthmatic mice exposed to different viral strains. These studies provide increased insight into secondary lung infections, but fail to address the concept of inert antigen exposure and asthma exacerbation.

The allergen challenge of all animals before the secondary virus infection was performed for two reasons. The first, to replicate human encounters with potential allergens. The second was to ensure that Th_2_ mediated inflammation including eosinophils, would be present to respond to the virus. Respiratory viral infection is known to induce AHR and increase the number of inflammatory cells recovered in the bronchoalveolar lavage of humans, regardless of their allergy or asthma status [32, 33]. As expected, in our model both sensitized and non-sensitized animals developed AHR, and had increased total numbers of inflammatory cells in the BAL. Only the sensitized group had significant increases in eosinophils compared to non-sensitized controls. In our model, we attribute the lack of response by non-sensitized animals to the UV-inactivated PIV to the lower presence of reactive Th_2_ inflammatory cells in the non-atopic airways, specifically eosinophils [34]. However, we acknowledge other allergic inflammatory cells could also play a significant role.

Sensitized animals treated with dexamethasone and re-exposed to live PIV or UV-inactivated PIV had significantly decreased AHR and inflammatory cell numbers in their BAL. This result was not unexpected, given the known effects of corticosteroids on lymphocytes and eosinophils, and their effects on AHR and inflammation in allergen challenged and virus infected animal models [35–37]. Although we did not confirm the exact cell type involved in the mechanism, this steroid response likely reflects the role of underlying allergic inflammation.

In order to study the response of allergic inflammation to virus antigen, we selected the guinea pig model. Guinea pigs have been described as “the premier model to study infections because of their similarities with regard to symptoms and immune response” [38]. Amongst other primary features, guinea pigs have similar pulmonary physiology and pharmacologic responses compared to humans [39–41]. The main detriment to our study using this model was the lack of commercially available immunohistochemical reagents (i.e., antibodies to identify specific cell subtype surface markers), preventing a detailed profiling of individual cell types involved in the inflammatory process. PIV was chosen as the respiratory viral pathogen for its known ability to infect both humans and guinea pigs, and its ability to induce asthma attacks in humans [1, 3, 42].

The best prophylactic treatment to prevent asthma attacks is anti-inflammatory therapy, typically inhaled corticosteroids. As most asthma attacks are linked to viruses, it would be logical to assume that corticosteroids are in some way decreasing inflammation before virus infection. This lowered inflammation could be responsible for a significant part of the improved response rather than the severity of virus infection. Our data supports the idea that improved prevention of virus-induced asthma exacerbations can occur through a better understanding of the interactions between viruses and inflammatory cells present in the airway before infection.

## Methods

### Animal ethics statement

All animals were used in accordance with the standards and protocols of the University of Alberta’s Animal Care and Use Committee for the Health Sciences (ACUC-HS, protocol number 061/03/06/D) and the guidelines of the Canada Council on Animal Care (CCAC).

### Animal housing and care

All animals were purchased from Charles River Canada and housed in a level 3 bio-containment facility at the University of Alberta. Animals were received at 1 month of age. Each animal was housed individually in a sealed bio-containment cage with its own independent air circulation. Animals were assessed multiple times per day by facility staff to ensure animals did not demonstrate physical signs of pain or stress such as hair loss, irritability, lethargy, and weight loss. Cages were lined with pine shavings and assessed regularly for cage cleanings, and ensuring ample water and food (Guinea Pig Diet 5025, LabDiet, St Louis, USA) was available to each animal.

### Guinea pig allergen sensitization

One month old, specific pathogen-free female Hartley guinea pigs (GP) were obtained and allowed to acclimatize to their new environment for 14 days (Fig 1). Animals were sensitized to ovalbumin via i.p. injections of 10 mg/kg of ultra-pure ovalbumin (Sigma-Aldrich, St Louis, MO, USA) dissolved in saline solution. A second i.p. injection of the same dose was given two days later. Sham injections were provided to additional guinea pigs consisting of NaCl 0.9% (Sigma-Aldrich, St Louis, MO, USA) following identical timelines. Sensitization and non-sensitization was confirmed in some animals using an i.v. injection of 10mg/kg of ultra-pure ovalbumin dissolved in saline [11].

### Parainfluenza virus culture

Parainfluenza (PIV) type 1 (Sendai virus, VR-105; American Type Culture Collection) was grown in cynomolgus monkey kidney cell (CMK) monolayers (ViroMed Laboratories) and stored in aliquots at −80°C. Inactivated PIV was created by exposing the virus to shortwave length (254nm) UV light for 30 minutes (UV-PIV). Virus inactivation was confirmed by the loss of virus-induced haemagluttination in CMK cells.

### Primary virus inoculation

Some GPs were inoculated intranasally with virus stock [11]. GPs were anesthetized intramuscularly with ketamine (30 mg/kg) and xylazine (5 mg/kg), and were inoculated intranasally with 0.5 mL of virus stock diluted in HBSS (Sigma-Aldrich) to produce a solution containing a 10^5^/mL tissue culture infectious dose (TCID50; 10^5^ times the concentration required to produce infection in 50% of rhesus monkey kidney monolayers). Sham inoculations consisted of intranasal administration of 0.5ml of HBSS. Infected and non-infected animals were separately housed in individually sealed cages with separate laminar airflow in a level 2 bio-containment facility.

### Aerosolized antigen challenge

On day 45 after recovery from virus or sham infection, all GPs were placed individually into a sealed chamber with one access port. The animals were exposed to an aerosolized solution of 0.5% ovalbumin (Sigma-Aldrich, St Louis, MO, USA) for 20 seconds. Ovalbumin sensitization was further confirmed with this treatment as only the ovalbumin sensitized GPs would develop visible respiratory distress (rapid respiratory rate and lethargy) after this challenge.

### Dexamethasone treatment

GPs on days 65 to 69 were given i.p. injections of 0.6 mg/kg dexamethasone dissolved in NaCl 0.9% (Sigma-Aldrich, St Louis, MO, USA) for 4 consecutive days before being re-exposed to live PIV or UV-inactivated PIV. Animals not selected to receive dexamethasone were given sham i.p. injections with NaCl 0.9%.

### Secondary virus inoculation

Similar methods for virus inoculation were performed again 60 days after virus infection. GPs were anesthetized intramuscularly with ketamine (30 mg/kg) and xylazine (5 mg/kg), and were inoculated intranasally with 0.5 mL of virus stock diluted in HBSS (Sigma-Aldrich) to produce a solution containing a 10^5^/mL tissue culture infectious dose (TCID50; 10^5^ times the concentration required to produce infection in 50% of rhesus monkey kidney monolayers). Sham inoculations consisted of intranasal administration of 0.5ml of HBSS. UV-inactivated virus was administered intranasally diluted with 0.5 mL of inactivated virus stock diluted in HBSS using the same dilution factor as the live virus stock.

### Assessment of airway hyperreactivity

All GPs underwent airway assessment 5 days after re-exposure to either sham, live or UV-inactivated PIV using a modified technique proposed by Dixon et al [43]. GP were anaesthetized tracheostomized and their external jugular vein was catheterized. GPs were tracheostomized, paralyzed with succinylcholine (10 g/kg/min. i.v., Sigma-Aldrich), and ventilated (tidal volume of 1 mL/100 g body weight; 100 BPM; Harvard Apparatus, South Natick, MA). Pulmonary inflation pressure (PIP) for each breath was recorded as a measure of bronchoconstriction at the tracheal cannula using a DTX pressure transducer and Powerlab software (AdInstruments, Colorado Springs, USA) [11]. GP were given doubling doses of histamine in 5-minute intervals (0.1–2.0 μg/kg i.v.) and the resulting change in pulmonary pressure was recorded [44]. Sensitization was re-confirmed after airway hyperreactivity measurements, with the I.V. injection of 10mg/kg of ovalbumin dissolved in NaCl 9% (Sigma-Aldrich, St Louis, MO, USA). At the end of the measurements, animals were euthanized with an overdose of urethane (3 g/kg i.v.) (Sigma-Aldrich, St Louis, MO, USA). Euthanasia with urethane was carried out according to the protocols approved by the ACUC-HS as dictated by CCAC guidelines [45].

### Bronchoalveolar lavage and cell staining

BAL was performed immediately after euthanization using 4–5 10 ml aliquots of 0.9% NaCl until 40mL of lavage fluid was collected on ice. The BAL was centrifuged at 300G for 10 minutes at 4°C. The pellet was resuspended in 1 mL of erythrocyte lysis solution (R&D Systems Inc, Minneapolis, MN, USA), and diluted with 9 mL of 0.9% NaCl. The cells were then centrifuged again at 300 g for 10 minutes, and a cell pellet was collected. The pellet was then resuspended in 10 mL of 0.9% NaCl. Total cell numbers were determined from a 10 μL sample pipetted onto a hemocytometer. 100,000 cells from lavage fluid were cytospun on to glass slides. The slides were then stained using a commercial staining solution (Diff-Quik, Baxter Healthcare Corp, Mississauga, Ontario, Canada). The slides were allowed to dry, then put into the fixative solution (containing fast green in methanol) for 30 seconds. After 30 seconds the solution was allowed to drain off the slide. The slide was next put into solution 1 (containing Eosin G in phosphate buffer) for 30 seconds. After 30 seconds the solution was allowed to drain off the slide. The slide was then put into solution 2 (thiazine dye in phosphate buffer) for 30 seconds. After 30 seconds had passed the slide was rinsed with distilled water and allowed to air dry. A slide coverslip was then glued over top of the slide using (Permount, Fisher Scientific, Ottawa, Ontario, Canada), and the cells were counted using light microscopy.

### Lung removal for histology

Lungs were removed immediately after BAL collection. A section of lung tissue (right upper lobe) was stored in an -80°C freezer for viral titer assessment. The remaining lung was re-inflated and fixed in 3.7% formaldehyde in 0.1% phosphate buffer (Sigma-Aldrich, St Louis, MO, USA) for 24 hours. After 24 hours the lungs were cut into 1.5 cm^2^ pieces placed in tissue cassettes and stored in 70% ethanol (Sigma-Aldrich, St Louis, MO, USA) at 4°C until fixed in paraffin and sectioned (6.0 μm).

### Measurement of virus memory using lymphocyte culture and BrdU incorporation assay

Methods were adapted from in vitro work isolating human lymphocytes [12]. The tracheobronchial lymph nodes are manually broken down under sterile conditions by using abrasive rubbing between glass slides in 10 mL of complete RPMI solution at 4°C. The lymph node homogenate solution was then pipetted up and down 10 times to remove adhered cells. The remaining tissue was removed by passing the homogenized sample through a 40 μm cell strainer (BD Biosciences, Canada). The resulting cell solution was centrifuged at 300 g for 10 minutes at 4°C, and the resulting cell pellet was resuspended in 10 mL of complete RPMI. The cell solution was then incubated in a 6-well plate for 2 hours at 37°C to remove adherent cells. The non-adherent cells were collected and the plates were lightly washed with room temperature RPMI to remove any remaining loosely adherent cells. The collected cells were then centrifuged in 10 mL of Ficoll (Pharmacia Biotech, Uppsala, Sweden) at room temperature. Lymphocyte bands were collected and resuspended in 1.5 mL of complete RPMI solution, and centrifuged at 300 g for 10 minutes at 4°C. The cells were resuspended in 10 mL of complete RPMI and were incubated for 45 minutes in a nylon wool column (Polyscience Inc, Warrington, PA, USA) to enrich for T cells. The enriched T cell solution was collected and cell viability was determined using trypan blue exclusion test. Cells were then cultured in a 96 well plate with 100,000 cells in 200 μL volume being plated per well. Viral memory responses were measured via a cell proliferation assay using BrdU incorporation (BrdU Cell Proliferation ELISA Chemiluminescent Kit; Roche, Mannheim, Germany) with the FLx800 luminometer (Biotek, Winooski, Vt) and determined as relative light units. Cells were cultured in complete RMPI solution with either BrdU alone (background luminescence), BrdU + phytohemagglutinin (Life Technologies Inc, Burlington, Ontario, Canada) (positive proliferation control), BrdU + ^1^/_1000_ dilution of Rhino Virus (RV type 16) (virus specific proliferation control), or BrdU + ^1^/_1000_ dilution of PIV stock solution (viral memory) with 0.2 μL added to the wells.

### Determination of virus titers

Confirmation of the absence of replicating virus for UV-virus inactivation was determined through analysis of lung samples (right upper lobe) from all animals re-exposed to UV-inactivated virus. Following airway physiology experiments, RNA was isolated from homogenized lung using RNAeasy mini kit (Qiagen, Toronto, Canada). Sample cDNA was generated use Superscript III Reverse Transcriptase and PCR was performed. PIV RNA was normalized to 18S and transformed to TCID_50_ units using a previously established PIV standard curve quantified by hemadsorption assay using rhesus monkey kidney cells.

### Lung histology and eosinophil infiltrate quantification

Lung sections preserved from each animal where stained with hematoxylin and eosin for determination of cellular infiltrate around large airways. On each slide three large cartilaginous airways were identified from different lobes. These airways were then examined using oil immersion light microscopy (100x magnification) and eosinophils were identified based on eosin uptake and morphology (bi-lobed nucleus, red staining granules) and 10 different visual fields around each airway counted for cell number. Eosinophils were identified in the airways, the mucosa, the submucosal space, and deep to the smooth muscle ending at the cartilaginous tissue surrounding the airway.

### Statistical analysis

T-cell proliferation data and eosinophil infiltrate data were analyzed using ANOVA with Dunnett’s multiple comparison test to identify statistical significance between the means. All other data were analyzed using Two-Way ANOVA with the Bonferroni multiple comparison test to identify statistical significance between the means. All data analyses were performed on GraphPad Prism version 6.0 software.

## References

[1] Johnston SL, Pattemore PK, Sanderson G, Smith S, Lampe F, Josephs L, et al Community study of role of viral infections in exacerbations of asthma in 9–11 year old children. BMJ. 1995;310(6989):1225–9. ; PubMed Central PMCID: PMCPMC2549614. 7767192 10.1136/bmj.310.6989.1225 PMC2549614

[2] Nicholson KG, Kent J, Ireland DC. Respiratory viruses and exacerbations of asthma in adults. BMJ. 1993;307(6910):982–6. ; PubMed Central PMCID: PMCPMC1679193. 8241910 10.1136/bmj.307.6910.982 PMC1679193

[3] Johnston SL, Pattemore PK, Sanderson G, Smith S, Campbell MJ, Josephs LK, et al The relationship between upper respiratory infections and hospital admissions for asthma: a time-trend analysis. Am J Respir Crit Care Med. 1996;154(3 Pt 1):654–60. doi: 10.1164/ajrccm.154.3.8810601 . 8810601 10.1164/ajrccm.154.3.8810601

[4] Winther B, McCue K, Ashe K, Rubino JR, Hendley JO. Environmental contamination with rhinovirus and transfer to fingers of healthy individuals by daily life activity. J Med Virol. 2007;79(10):1606–10. doi: 10.1002/jmv.20956 . 17705174 10.1002/jmv.20956

[5] Steinke JW, Liu L, Turner RB, Braciale TJ, Borish L. Immune surveillance by rhinovirus-specific circulating CD4+ and CD8+ T lymphocytes. PLoS One. 2015;10(1):e0115271 doi: 10.1371/journal.pone.0115271 ; PubMed Central PMCID: PMCPMC4293146. 25584821 10.1371/journal.pone.0115271 PMC4293146

[6] Bel EH. Clinical phenotypes of asthma. Curr Opin Pulm Med. 2004;10(1):44–50. . 14749605 10.1097/00063198-200401000-00008

[7] Hamid Q, Tulic MK, Liu MC, Moqbel R. Inflammatory cells in asthma: mechanisms and implications for therapy. The Journal of allergy and clinical immunology. 2003;111(1 Suppl):S5–S12; discussion S-7. . 12532083 10.1067/mai.2003.22

[8] Hastie AT, Moore WC, Meyers DA, Vestal PL, Li H, Peters SP, et al Analyses of asthma severity phenotypes and inflammatory proteins in subjects stratified by sputum granulocytes. The Journal of allergy and clinical immunology. 2010;125(5):1028–36 e13. doi: 10.1016/j.jaci.2010.02.008 ; PubMed Central PMCID: PMCPMC2878277. 20398920 10.1016/j.jaci.2010.02.008 PMC2878277

[9] Jacoby DB, Gleich GJ, Fryer AD. Human eosinophil major basic protein is an endogenous allosteric antagonist at the inhibitory muscarinic M2 receptor. The Journal of clinical investigation. 1993;91(4):1314–8. doi: 10.1172/JCI116331 ; PubMed Central PMCID: PMCPMC288101. 8473484 10.1172/JCI116331 PMC288101

[10] Barnes PJ. Anti-inflammatory actions of glucocorticoids: molecular mechanisms. Clin Sci (Lond). 1998;94(6):557–72. . 9854452 10.1042/cs0940557

[11] Adamko DJ, Yost BL, Gleich GJ, Fryer AD, Jacoby DB. Ovalbumin sensitization changes the inflammatory response to subsequent parainfluenza infection. Eosinophils mediate airway hyperresponsiveness, m(2) muscarinic receptor dysfunction, and antiviral effects. J Exp Med. 1999;190(10):1465–78. Epub 1999/11/24. ; PubMed Central PMCID: PMC2195693. 10562321 10.1084/jem.190.10.1465 PMC2195693

[12] Davoine F, Cao M, Wu Y, Ajamian F, Ilarraza R, Kokaji AI, et al Virus-induced eosinophil mediator release requires antigen-presenting and CD4+ T cells. The Journal of allergy and clinical immunology. 2008;122(1):69–77, e1-2. doi: 10.1016/j.jaci.2008.03.028 . 18472150 10.1016/j.jaci.2008.03.028

[13] Ilarraza R, Wu Y, Skappak CD, Ajamian F, Proud D, Adamko DJ. Rhinovirus has the unique ability to directly activate human T cells in vitro. The Journal of allergy and clinical immunology. 2013;131(2):395–404. doi: 10.1016/j.jaci.2012.11.041 . 23374267 10.1016/j.jaci.2012.11.041

[14] Barnes PJ. Glucocorticoids. Chem Immunol Allergy. 2014;100:311–6. doi: 10.1159/000359984 . 24925411 10.1159/000359984

[15] Harvey AM, Howard JE, Winkenwerder WL, Bordley JE, Carey RC, Kattus A. Observations on the Effect of Adrenocorticotrophic Hormone (ACTH) on Disseminated Lupus Erythematosus, Drug Hypersensitivity Reactions, and Chronic Bronchial Asthma. Trans Am Clin Climatol Assoc. 1949;61:221–8. ; PubMed Central PMCID: PMCPMC2241996. 21407721 PMC2241996

[16] Chu EK, Drazen JM. Asthma: one hundred years of treatment and onward. Am J Respir Crit Care Med. 2005;171(11):1202–8. doi: 10.1164/rccm.200502-257OE . 15778490 10.1164/rccm.200502-257OE

[17] Sly PD, Boner AL, Bjorksten B, Bush A, Custovic A, Eigenmann PA, et al Early identification of atopy in the prediction of persistent asthma in children. Lancet. 2008;372(9643):1100–6. doi: 10.1016/S0140-6736(08)61451-8 ; PubMed Central PMCID: PMCPMC4440493. 18805338 10.1016/S0140-6736(08)61451-8 PMC4440493

[18] Wark PA, Johnston SL, Bucchieri F, Powell R, Puddicombe S, Laza-Stanca V, et al Asthmatic bronchial epithelial cells have a deficient innate immune response to infection with rhinovirus. J Exp Med. 2005;201(6):937–47. Epub 2005/03/23. doi: 10.1084/jem.20041901 ; PubMed Central PMCID: PMC2213100. 15781584 10.1084/jem.20041901 PMC2213100

[19] Denlinger LC, Sorkness RL, Lee WM, Evans MD, Wolff MJ, Mathur SK, et al Lower airway rhinovirus burden and the seasonal risk of asthma exacerbation. Am J Respir Crit Care Med. 2011;184(9):1007–14. doi: 10.1164/rccm.201103-0585OC ; PubMed Central PMCID: PMCPMC3208645. 21816938 10.1164/rccm.201103-0585OC PMC3208645

[20] Contoli M, Message SD, Laza-Stanca V, Edwards MR, Wark PA, Bartlett NW, et al Role of deficient type III interferon-lambda production in asthma exacerbations. Nat Med. 2006;12(9):1023–6. doi: 10.1038/nm1462 . 16906156 10.1038/nm1462

[21] Bardin PG, Fraenkel DJ, Sanderson G, Dorward M, Lau LC, Johnston SL, et al Amplified rhinovirus colds in atopic subjects. Clinical and experimental allergy: journal of the British Society for Allergy and Clinical Immunology. 1994;24(5):457–64. Epub 1994/05/01. . 8087657 10.1111/j.1365-2222.1994.tb00934.x PMC7164826

[22] Kennedy JL, Shaker M, McMeen V, Gern J, Carper H, Murphy D, et al Comparison of viral load in individuals with and without asthma during infections with rhinovirus. Am J Respir Crit Care Med. 2014;189(5):532–9. doi: 10.1164/rccm.201310-1767OC ; PubMed Central PMCID: PMCPMC3977713. 24471509 10.1164/rccm.201310-1767OC PMC3977713

[23] Kato M, Ishioka T, Kita H, Kozawa K, Hayashi Y, Kimura H. Eosinophil granular proteins damage bronchial epithelial cells infected with respiratory syncytial virus. Int Arch Allergy Immunol. 2012;158 Suppl 1:11–8. doi: 10.1159/000337752 . 22627361 10.1159/000337752

[24] Fujimoto K, Kubo K, Matsuzawa Y, Sekiguchi M. Eosinophil cationic protein levels in induced sputum correlate with the severity of bronchial asthma. Chest. 1997;112(5):1241–7. . 9367463 10.1378/chest.112.5.1241

[25] Alfaro C, Sharma OP, Navarro L, Glovsky MM. Inverse correlation of expiratory lung flows and sputum eosinophils in status asthmaticus. Annals of allergy. 1989;63(3):251–4. . 2774309

[26] Gibson PG, Simpson JL, Hankin R, Powell H, Henry RL. Relationship between induced sputum eosinophils and the clinical pattern of childhood asthma. Thorax. 2003;58(2):116–21. ; PubMed Central PMCID: PMCPMC1746565. doi: 10.1136/thorax.58.2.116 12554891 10.1136/thorax.58.2.116 PMC1746565

[27] Adamko DJ, Fryer AD, Bochner BS, Jacoby DB. CD8+ T lymphocytes in viral hyperreactivity and M2 muscarinic receptor dysfunction. Am J Respir Crit Care Med. 2003;167(4):550–6. Epub 2002/11/02. doi: 10.1164/rccm.200206-506OC . 12411283 10.1164/rccm.200206-506OC

[28] Davoine F, Cao M, Wu Y, Ajamian F, Ilarraza R, Kokaji AI, et al Virus-induced eosinophil mediator release requires antigen-presenting and CD4+ T cells. The Journal of allergy and clinical immunology. 2008;122(1):69–77, e1-2. Epub 2008/05/13. doi: 10.1016/j.jaci.2008.03.028 . 18472150 10.1016/j.jaci.2008.03.028

[29] Culley FJ, Pollott J, Openshaw PJ. Age at first viral infection determines the pattern of T cell-mediated disease during reinfection in adulthood. The Journal of experimental medicine. 2002;196(10):1381–6. Epub 2002/11/20. ; PubMed Central PMCID: PMC2193991. doi: 10.1084/jem.20020943 12438429 10.1084/jem.20020943 PMC2193991

[30] Lee YM, Miyahara N, Takeda K, Prpich J, Oh A, Balhorn A, et al IFN-gamma production during initial infection determines the outcome of reinfection with respiratory syncytial virus. American journal of respiratory and critical care medicine. 2008;177(2):208–18. Epub 2007/10/27. doi: 10.1164/rccm.200612-1890OC ; PubMed Central PMCID: PMC2204078. 17962634 10.1164/rccm.200612-1890OC PMC2204078

[31] Furuya Y, Roberts S, Hurteau GJ, Sanfilippo AM, Racine R, Metzger DW. Asthma increases susceptibility to heterologous but not homologous secondary influenza. J Virol. 2014;88(16):9166–81. doi: 10.1128/JVI.00265-14 ; PubMed Central PMCID: PMCPMC4136262. 24899197 10.1128/JVI.00265-14 PMC4136262

[32] Laitinen LA, Kava T. Bronchial reactivity following uncomplicated influenza A infection in healthy subjects and in asthmatic patients. European journal of respiratory diseases Supplement. 1980;106:51–8. Epub 1980/01/01. . 6937355

[33] Calhoun WJ, Dick EC, Schwartz LB, Busse WW. A common cold virus, rhinovirus 16, potentiates airway inflammation after segmental antigen bronchoprovocation in allergic subjects. The Journal of clinical investigation. 1994;94(6):2200–8. Epub 1994/12/01. doi: 10.1172/JCI117581 ; PubMed Central PMCID: PMC330045. 7989575 10.1172/JCI117581 PMC330045

[34] Wissinger EL, Stevens WW, Varga SM, Braciale TJ. Proliferative expansion and acquisition of effector activity by memory CD4+ T cells in the lungs following pulmonary virus infection. Journal of immunology. 2008;180(5):2957–66. Epub 2008/02/23. ; PubMed Central PMCID: PMC2855534. 18292518 10.4049/jimmunol.180.5.2957 PMC2855534

[35] Meagher LC, Cousin JM, Seckl JR, Haslett C. Opposing effects of glucocorticoids on the rate of apoptosis in neutrophilic and eosinophilic granulocytes. Journal of immunology. 1996;156(11):4422–8. Epub 1996/06/01. . 8666816

[36] Evans CM, Jacoby DB, Fryer AD. Effects of dexamethasone on antigen-induced airway eosinophilia and M(2) receptor dysfunction. American journal of respiratory and critical care medicine. 2001;163(6):1484–92. Epub 2001/05/24. doi: 10.1164/ajrccm.163.6.2007047 . 11371422 10.1164/ajrccm.163.6.2007047

[37] Moreno L, Jacoby DB, Fryer AD. Dexamethasone prevents virus-induced hyperresponsiveness via multiple mechanisms. American journal of physiology Lung cellular and molecular physiology. 2003;285(2):L451–5. Epub 2003/04/30. doi: 10.1152/ajplung.00046.2003 . 12716653 10.1152/ajplung.00046.2003

[38] Padilla-Carlin DJ, McMurray DN, Hickey AJ. The guinea pig as a model of infectious diseases. Comp Med. 2008;58(4):324–40. ; PubMed Central PMCID: PMCPMC2706043. 18724774 PMC2706043

[39] Partanen M, Laitinen A, Hervonen A, Toivanen M, Laitinen LA. Catecholamine- and acetylcholinesterase-containing nerves in human lower respiratory tract. Histochemistry. 1982;76(2):175–88. . 7161145 10.1007/BF00501920

[40] Canning BJ, Mazzone SB, Meeker SN, Mori N, Reynolds SM, Undem BJ. Identification of the tracheal and laryngeal afferent neurones mediating cough in anaesthetized guinea-pigs. The Journal of physiology. 2004;557(Pt 2):543–58. doi: 10.1113/jphysiol.2003.057885 ; PubMed Central PMCID: PMCPMC1665106. 15004208 10.1113/jphysiol.2003.057885 PMC1665106

[41] Kummer W, Fischer A, Kurkowski R, Heym C. The sensory and sympathetic innervation of guinea-pig lung and trachea as studied by retrograde neuronal tracing and double-labelling immunohistochemistry. Neuroscience. 1992;49(3):715–37. . 1380140 10.1016/0306-4522(92)90239-x

[42] Blomqvist GA, Martin K, Morein B. Transmission pattern of parainfluenza 3 virus in guinea pig breeding herds. Contemp Top Lab Anim Sci. 2002;41(4):53–7. . 12109899

[43] Dixon WE. Contributions to the physiology of the lungs: Part I. The bronchial muscles, their innervation, and the action of drugs upon them. The Journal of physiology. 1903;29(2):97–173. Epub 1903/03/16. ; PubMed Central PMCID: PMC1540618. 16992663 10.1113/jphysiol.1903.sp000947 PMC1540618

[44] Drazen JM, Austen KF. Effects of intravenous administration of slow-reacting substance of anaphylaxis, histamine, bradykinin, and prostaglandin F2alpha on pulmonary mechanics in the guinea pig. J Clin Invest. 1974;53(6):1679–85. Epub 1974/06/01. doi: 10.1172/JCI107719 ; PubMed Central PMCID: PMC302664. 4830230 10.1172/JCI107719 PMC302664

[45] Committee CReaobottahsoeotCCoACCG. CCAC guidelines on: euthanasia of animals used in science. CCAC Guidelines. 2010;1(1):1–32.

